# The Collective Benefits of Feeling Good and Letting Go: Positive Emotion and (dis)Inhibition Interact to Predict Cooperative Behavior

**DOI:** 10.1371/journal.pone.0117426

**Published:** 2015-01-27

**Authors:** David G. Rand, Gordon Kraft-Todd, June Gruber

**Affiliations:** 1 Department of Psychology, Yale University, New Haven, Connecticut, United States of America; 2 Department of Economics, Yale University, New Haven, Connecticut, United States of America; 3 School of Management, Yale University, New Haven, Connecticut, United States of America; 4 Department of Psychology and Neuroscience, University of Colorado Boulder, Boulder, Colorado, United States of America; University of Maribor, SLOVENIA

## Abstract

Cooperation is central to human existence, forming the bedrock of everyday social relationships and larger societal structures. Thus, understanding the psychological underpinnings of cooperation is of both scientific and practical importance. Recent work using a dual-process framework suggests that intuitive processing can promote cooperation while deliberative processing can undermine it. Here we add to this line of research by more specifically identifying deliberative and intuitive processes that affect cooperation. To do so, we applied automated text analysis using the Linguistic Inquiry and Word Count (LIWC) software to investigate the association between behavior in one-shot anonymous economic cooperation games and the presence inhibition (a deliberative process) and positive emotion (an intuitive process) in free-response narratives written after (Study 1, *N* = 4,218) or during (Study 2, *N* = 236) the decision-making process. Consistent with previous results, across both studies inhibition predicted reduced cooperation while positive emotion predicted increased cooperation (even when controlling for negative emotion). Importantly, there was a significant interaction between positive emotion and inhibition, such that the most cooperative individuals had high positive emotion and low inhibition. This suggests that inhibition (i.e., reflective or deliberative processing) may undermine cooperative behavior by suppressing the prosocial effects of positive emotion.

## Introduction

Cooperation plays an integral role in our lives, sustaining friendships and business relationships and laying the foundation for successful organizations and nations [1–18]. When people cooperate, they can achieve more than each could working alone: cooperation creates benefit, and is positively non-zero sum. Yet cooperation often requires individuals to bear personal costs in order to create those benefits, creating a social dilemma where individual and collective interests are in conflict. Given the societal benefits of cooperation, understanding the psychological underpinnings of cooperative behavior is of both scientific and practical importance [19–33]. Thus, it is critical to identify potential processes that may influence cooperative behavior.

A growing literature uses economic games to explore the cognitive underpinnings of cooperation from a dual process perspective. In these experiments, participants choose between keeping money for themselves or spending money to benefit others (cf. [34, 35]). For example, consider the following Public Goods Game (PGG): each member of a group of four starts with a $10 endowment, and decides how much to keep versus contribute to a “common project”. All contributions are then doubled and split evenly among the four group members. Contributing is individually costly regardless of the actions of the other group members (each dollar contributed is doubled and split four ways, so for every dollar you contribute you get back only $0.50). Yet if everyone contributes, everyone doubles their money. To understand the cognitive underpinnings of cooperation in such games, a dual process perspective is often employed, whereby decisions are conceptualized as resulting from the competition between two cognitive systems: one that is fast, automatic, intuitive and often emotional, and another that is slow, controlled, and deliberative [36–42]. Turning this dual process lens to cooperation raises the following question: Are people by default selfish, only acting cooperatively through inhibition and self-control? Or do we have automatic (perhaps emotional) impulses to cooperate, which are undermined by selfish deliberation?

A series of recent experiments support the latter possibility; namely, that time pressure [43–47], cognitive load [48–50], conceptual priming of intuition [44, 51], deciding about present rather than future allocations [52, 53], and disruption of the right lateral prefrontal cortex [54] can increase participants’ willing to pay money in economic games to benefit others (although some studies also find null effects [55–58]). Further evidence comes from studies find that participants project cooperative frames onto neutrally framed economic games [59]; that behavior in one-shot games is influenced by previous play of long versus short repeated games, but only for participants who rely on heuristics [60]; the priming intuition increases charitable donations to identifiable but not statistical victims [61]; that people with low self-control are more likely to sacrifice to benefit their romantic partners [62]; and that people who risk their lives to save strangers overwhelmingly describe their decision-processes as automatic and intuitive [63].

Yet the particular elements of deliberation and intuition that affect cooperative behavior remain unclear. Here we examine this issue. To guide our investigation, we turn to the *Social Heuristics Hypothesis* (SHH), which has recently been proposed as a mechanistic explanation of the above findings [43]. The SHH adds an explicitly dual-process perspective to theories of cultural evolution and norm internalization [64–67], and suggests that strategies which are typically successful in daily social life get automatized as social intuitions. When in atypical settings (such as one-shot interactions without future consequences), deliberation can then over-ride these automatic responses in favor of responses which are better tailored to the task at hand (selfishness, in the context of one-shot interactions).

Based on this model, a clear candidate for a specific deliberative process undermining cooperation is *inhibition*. Because daily life interactions typically involve future consequences [4, 10] many people acquire cooperative defaults, which are then inhibited by deliberation when people find themselves in interaction without future consequences. Therefore, we predict that inhibition will be associated with reduced cooperation.

Turning from deliberative processes to intuitive processes, a promising candidate is emotion, and in particular *positive emotion*. Emotional influences on prosocial behavior such as cooperation are well-documented, and positive emotion in particular is a critical component for adaptive social functioning and associated with many salutary social effects relevant to cooperation [68–70]. For example, a core function of positive emotion is to provide fertile ground to build and maintain vital social resources to function within a larger group structure [71]. For example, positive feelings provide meaning and enjoyment when forming social alliances [71–73] and help foster relationship satisfaction and commitment [74, 75], and promote increased prosocial behaviors necessary for cooperation, including helping others [76, 77]. Finally, greater self-reported positive emotion levels have been associated with an individual’s ability to understand others’ emotions, a critical skill in building cooperative ties with others [78, 79]. These beneficial social effects of positive emotion suggest that feeling positive might directly influence the extent to which an individual cooperates with others. Consistent with this, gratitude as a critical positive emotion has been shown to promote cooperation in economic exchanges [80]. Therefore, we predict that positive emotion will be associated with increased cooperation.

Furthermore, we predict an interaction between inhibition and positive emotion. Specifically, if positive emotion is a key component of intuitive processing that favors cooperation, and deliberation impairs cooperation through the reigning-in of positive emotion, then the negative effect of inhibition should be greater when more positive emotion is present (and the positive effect of positive emotion should be reduced when inhibition is present). To test these predictions, we predict behavior in economic cooperation games using the presence of positive emotion and inhibition (quantified using the well-validated Linguistic Inquiry and Word Count tool [81], LIWC; see below for further details) in free-response narratives spontaneously generated after (Study 1, *N* = 4,218) or during (Study 2, *N* = 236) the cooperative decision-making process.

## Study 1

In Study 1, we investigated the role positive emotion and inhibition play in cooperation by analyzing participants’ free response descriptions of their decision-making process in a one-shot economic cooperation game, written after finishing the game.

### Participants

Participants were 4,218 adult U.S, residents (44.9% female, *M*_age_ 31.0 years [SD = 11.05 yrs], median education level “Attended College”) drawn from nine different social dilemma studies run on Amazon Mechanical Turk [82–86] between January 2011 and January 2013 in which free-response narratives were collected but not previously analyzed. These studies were approved by the Yale University Human Subjects Committee IRB Protocol #1307012383. All subjects provided written informed consent prior to participating, and this was approved by the Human Subjects Committee. See S1 Dataset for raw data.

### Cooperation Task

Seven studies involved a one-shot Public Goods Game where groups of four participants chose how much of $0.40 to keep and how much to contribute to a common project, with contributions be doubled and split equally among the four group members. The other two involved a one-shot continuous (rather than binary) Prisoner’s Dilemma, which is a two-player analog of the PGG: each subject chose how much of $0.40 to keep and how much to transfer to the other, with transfers being doubled. Each study involved two or more experimental conditions, which we aggregate for the present analyses. These economic games are well established as measures of cooperation [4, 34], and have been used previously to specifically examine the role of intuition versus deliberation in cooperation [43–46]. Earnings in all experiments were determined by game play, and no deception was used.

After making their decision in the game, participants’ comprehension of the game payoff structure was assessed by asking: “What level of contribution earns the highest payoff for the group as a whole?” and “What level of contribution earns the highest payoff for you personally?”, and were informed that they had to answer correctly in order to get paid (27.5% of subjects that answered one or both questions incorrectly—most errors involved the incorrect belief that cooperation was individually optimal). Subjects who failed the comprehension check may not have understood that they were facing a social dilemma, and thus it is not clear whether their behavior is actually “cooperative”. Therefore our analyses include a check for whether comprehension interacts with our variables of interest.

### Quantitative Analysis of Emotion and Inhibition

At the end of the study, participants provided free-response answers to the prompt “describe why you made your decision in the game” (Length of Response: *M* = 17.51 words, *SD* = 11.67), from which we performed a quantitative text analysis using the Linguistic Inquiry and Word Count (LIWC). LIWC is a computerized text analysis program that counts the frequencies of words which have been demonstrated to represent different psychologically relevant categories [81]. Specifically, LIWC scans the words of a text document against its internal dictionary that contains over 70 categories, and assigns each word into a specific category. It then outputs the percentage of words in the document belonging to each category [87]. LIWC has been widely used to track naturally-occurring behavior and language use across a variety of contexts, including classical literature, press conferences, everyday conversations, and personal narratives (e.g. [88, 89]). Moreover, the LIWC allows for automated and efficient coding of features of verbal behavior that may be less detectable to even a highly trained team of human coders. Thus, carefully attending to the specific words people use during naturalistically occurring narratives can be reliably coded using the LIWC.

Of particular relevance for the current work, we derived the presence of words in three theoretically relevant LIWC categories; specifically, we focused on word frequencies related to positive emotion (“positive emotions” category, including words such as “love,” “nice,” and “sweet”), reflective processing (“inhibition” category, including words such as “block,” “constrain,” and “stop”), as well as negative emotion (“negative emotions” category, including words such as “hurt,” “ugly,” and “nasty”) as a control. These three affective and cognitive LIWC categories have been widely used in previous LIWC studies [63, 90–92].

To provide further insight into the LIWC classification, sample PGG narratives that received high LIWC scores for each of these three categories are shown in Table 1. We see that texts classified into each category are broadly consistent with the relevant concepts, even if the mapping is not perfect. We follow the procedure of [63], and focus our analysis how the presence of these three categories of words (0 = absent, 1 = present) predicted subsequent cooperation in the game (the distribution of LIWC scores was very right-skewed, making a continuous rather than binary analysis less meaningful).

**Table 1:** Example texts that received high LIWC scores for positive emotion, negative emotion, and inhibition.

**Positive Emotion**	**Negative Emotion**	**Inhibition**
Best benefits everyone	I am selfish, and I’m sorry :(	seemed safest to keep my money
I like sharing with others.	I am a timid person and risk-averse.	It was the safest and most secure decision.
Giving and sharing makes me happier.	risk…..not all was risked, but I wasn’t selfish with it either.	keep some, share some
I like to see everyone win	best outcome w/o greed	I decided to play it safe, and keep my money
something like a game.	Neither profit nor loss	It was the safest bet
I like my chances better that way.	I apparently felt greedy.	tried to keep the most
I like my shares to be fair	I did not want to risk losing money	It felt like a safe amount.
Because I enjoy helping people.	I didn’t want to risk losing that much	It seemed like a safe bet

Shown are the texts from Study 1 rated most highly in each category. For this table (but not our analyses) we restrict to texts containing over 20 characters for greater interpretability.

## Results

**Fig 1:**
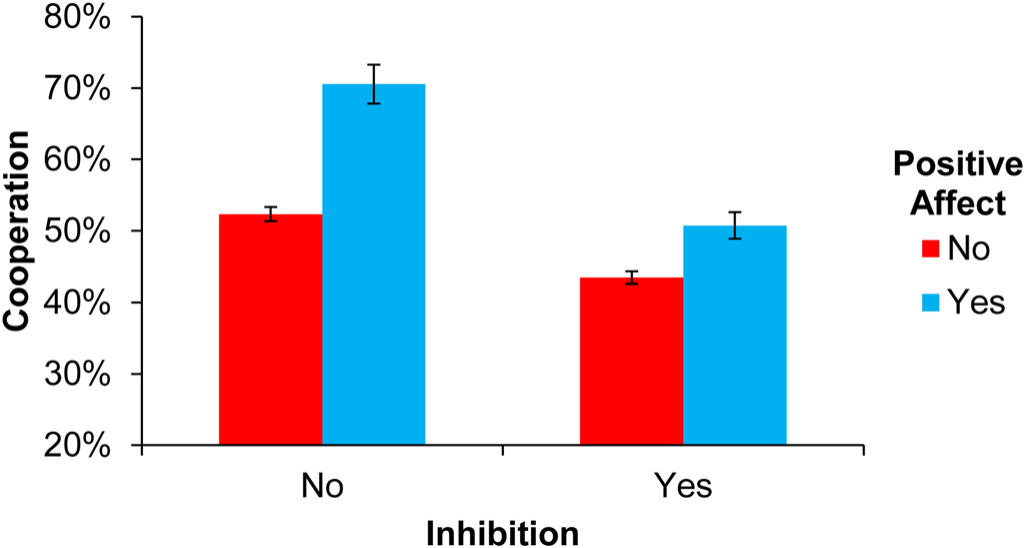
Fraction of endowment spent on cooperation in Study 1. Error bars indicate standard errors of the mean.

As seen in Fig. 1, participants indicating positive emotion were more cooperation than those who did not mention positive emotion, while those who mentioned inhibition cooperated less than those who did not. Furthermore, the presence of inhibition substantially diluted the effect of positive emotion. An ANOVA predicting cooperation confirmed this visual impression, finding a significant main effect of Positive Emotion [*F*(1,4214) = 45.02, *p*<0.001], a significant main effect of Inhibition [*F*(1,4214) = 56.97, *p*<0.001], and a significant interaction between Positive Emotion and Inhibition [*F*(1,4214) = 8.31, *p*<0.01]. Examining simple effects, we found a significant effect of Positive Emotion in participants both with [*F*(1,522) = 2.22, *p*<0.05] and without [*F*(1,3692) = 14.1, *p*<0.0001] Inhibition, and a significant negative effect of Inhibition in participants both with [*F*(1,2473) = 9.08, *p*<0.0001] and without [*F*(1,1741) = 2.82, *p*<0.005] Positive Emotion.

Importantly, these results were robust to controlling for the presence (or absence) of Negative Emotion, as well as age (continuous), gender, education (categorical), and total word count (continuous) (Positive Emotion [*F*(1,4204) = 39.86, *p*<0.001], Inhibition [*F*(1,4204) = 58.91, *p*<0.001], Positive Emotion x Inhibition interaction [*F*(1,4204) = 6.76, *p*<0.01]. The robustness of our results for Positive Emotion when controlling for Negative Emotion suggests that it was truly the presence of positive emotion, rather than the absence of negative emotion, that drove our findings.

We also note a lack of significant interaction between our variables of interest and comprehension of the game payoff structure (Comprehension x Positive Emotion [*F*(1,4209) = 0.55, *p* = 0.460], Comprehension x Inhibition [*F*(1,4209) = 2.22, *p* = 0.136], Comprehension x Positive Emotion x Inhibition [*F*(1,4209) = 0.00, *p* = 0.981]), as well as the fact that our results persist when excluding participants who answered any questions incorrectly (without controls: PE [*F*(1,3,051) = 40.72, *p*<0.001], Inhibition [*F*(1,3,051) = 29.09, *p*<0.001], Positive Emotion x Inhibition [*F*(1,3051) = 7.12, *p*<0.01]; with controls: Positive Emotion [*F*(1,3,041) = 35.94, *p*<0.001], Inhibition [F(1,3041) = 31.11, p<0.001], Positive Emotion x Inhibition [*F*(1,3041) = 5.84, *p*<0.05]).

Finally, although the effect of Negative Emotion was not the focus of this investigation, for completeness we report results for Negative Emotion separately, adopting a parallel analytic approach as for Positive Emotion. These results did not yield a significant main effect of Negative Emotion [F(1,4214) = 0.51, p = .475], but did yield a significant main effect of Inhibition [F(1,4214) = 22.64, p<0.001], and a significant interaction of Negative Emotion x Inhibition [F(1,4214) = 14.20, p<0.001]; leading to a significant negative simple effect of Negative Emotion without Inhibition [F(1,4214) = 33.59, p<0.001], and a non-significant but trending positive effect of Negative Emotion with Inhibition [F(1,4214) = 3.38, p = 0.066].

## Study 2

In Study 2, we further investigated the role positive emotion and inhibition play in cooperation using stream-of-consciousness narratives written *during* a one-shot PGG, rather than afterwards, as participants’ post-decision recollections of their decision-making process may be biased. These studies were approved by the Yale University Human Subjects Committee IRB Protocol #1307012383. All subjects provided written informed consent prior to participating, and this was approved by the Human Subjects Committee. See S2 Dataset for raw data.

### Participants

Participants were 236 adult US residents (41.8% female, *M*_age_ 30.7 years [SD = 10.64 yrs], median education level “Attended College”) recruited using Amazon Mechanical Turk in January 2013.

### Cooperation Task

Participants played the same PGG described in Study 1 (groups of 4, $0.40 endowment, contributions multiplied by 2), but with comprehension assessed before making their decision rather than afterwards (22.0% of participants answered one or both questions incorrectly). To elicit participants’ motivations *during* their decision, rather than afterwards as in Study 1, they were given the prompt “While you are considering your decision, please type your thoughts as they occur to you” along with a corresponding text box, immediately above a series of radio buttons used to entered their contribution choice. We wanted participants to record enough of their thoughts for us to have sufficient signal for our analyses, but we were also concerned that mandating a minimum length might affect the decision making process. Thus we randomized subjects into conditions with a minimum of 0, 10, 30, or 60 seconds of thinking. This allowed us to generate sufficient signal, while also assessing any potential effects of mandating certain writing time periods.

### Quantitative Analysis of Emotion and Inhibition

Participants’ free-response narratives were analyzed using the same LIWC software as in Study 1.

## Results

**Fig 2:**
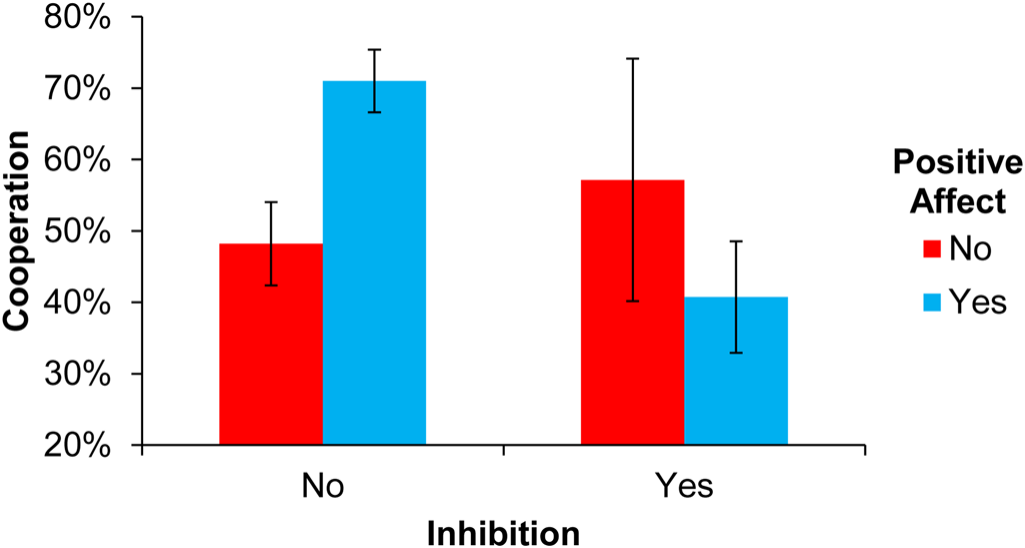
Fraction of endowment spent on cooperation in Study 2. Error bars indicate standard errors of the mean.

Preliminary analyses found a significant three-way interaction between Positive Emotion, Inhibition and Comprehension failure [*F*(1,228) = 4.67, *p*<0.05]. To avoid bias introduced by misunderstanding the game structure, we therefore focused our analysis on the 184 participants that answered the comprehension questions correctly. As seen in Fig. 2, we did not find a main effect for Positive Emotion [*F*(1,180) = 0.11, *p* = 0.75] or Inhibition [*F*(1,180) = 1.20, *p* = 0.28]. Similarly to Study 1, however, we found a significant Positive Emotion x Inhibition interaction [*F*(1,180) = 4.05, *p*<0.05] such that positive emotion increased cooperation, but this effect was suppressed by Inhibition. Examining simple effects, we found a significant effect of Positive Emotion in participants without Inhibition [*F*(1,148) = 3.14, *p*<0.005] but not with Inhibition [*F*(1,32) = 0.93, *p* = 0.36]; and a significant negative effect of Inhibition in participants with Positive Emotion [*F*(1,119) = 3.30, *p*<0.005] but not without Positive Emotion [*F*(1,61) = 0.51, *p* = 0.61].

As in Study 1, these results were robust to controlling for the presence of Negative Emotion, as well as age (continuous), gender, education (categorical), total word count of the description (continuous) and minimum writing time (categorical) (Positive Emotion [*F*(1,162) = 0.05, *p* = .822], Inhibition [*F*(1,162) = 1.98, *p* = 0.161], Positive Emotion x Inhibition [*F*(1,162) = 4.24, *p*<0.05]), again indicating that it is truly the presence of positive emotion, rather than the absence of negative emotion, that drives the observed results.

Finally, for completeness we describe parallel analyses for Negative Emotion, which did not find a significant main effect of Negative Emotion [F(1,232) = 0.46, p = .498] or a significant Negative Emotion x Inhibition interaction [F(1,232) = 2.36, p = 0.126], but did find a significant main effect of Inhibition [F(1,232) = 7.56, p<0.001].

## Discussion

Across two studies, we provided evidence that positive emotion motivates cooperation, that inhibition undermines cooperation, and that these two processes interact: positive emotion without inhibition was associated with the highest level of cooperation. This interaction between positive emotion and inhibition helps to provide a mechanism underlying previous results on the negative consequences of deliberation for cooperation: deliberation may undermine cooperative behavior by dampening the socially beneficial effects of positive emotion.

We build on previous work related to positive emotion and prosociality [68, 69, 71, 73] by focusing on an objective and quantitative analysis of naturally occurring emotional behavior, as opposed to deriving measures of emotion from self-reported indices subject to demand characteristics and inherent biases in questionnaire measurement of emotion responding. By combining analysis of narrative text with play in economic games for the first time, we bridge between different experimental traditions, and shed light on actual (rather than hypothetical) decision-making. The positive link our work suggests between positive emotion and cooperation is consistent with evidence that inducing gratitude increases cooperation [93], and more generally suggests the importance of manipulations that can promote positive emotions with salutary social effects such as compassion [24] and elevation [94, 95].

Our use of automated text analysis allowed us to quantitatively represent the emotional content of our participants’ narratives, and avoids potential coder biases. Our results linking inhibitory language to selfishness are consistent with work analyzing the testimony of Carnegie Heroes, people who risked their lives to save strangers [63]. The same LIWC algorithm used here found much less inhibitory language in the heroes’ descriptions of their decision process compared to deliberative control statements, suggesting that altruistic action requires lack of inhibition. Our LIWC results are also consistent with another text analysis of post-game narratives which looked for words that were significantly more common among one group of participants compared to another [96], and found that participants primed to be more intuitive were more likely to use words such as “feel”, “good”, “hope”, and “give”, and that use of these words in turn predicted cooperation.

The present results should be interpreted within the confines of several limitations. First, we note that our measures of positive (and negative) emotion were obtained at a general valence level, which precluded us from providing insight into the *specific* positive emotions involved in cooperation. Exploring this issue is an important direction for future work, given divergent associations between self-focused emotion such as pride and other-focused emotions like compassion on social intuitions [97]. Secondly, our measures were obtained using the default LIWC dictionaries. Although these dictionaries have been previously validated [98], their definitions of positive emotion and inhibition may not match perfectly with how these constructs are used in the fields relevant for the present research. Thus future work might explore the effect of analyzing narratives using custom designed dictionaries.

## Supporting Information

S1 Dataset Raw data in csv format for Study 1. (CSV)

S2 Dataset Raw data in csv format for Study 2. (CSV)
